# Supplementary material for: Splice-Junction-Based Mapping of Alternative Isoforms in the Human Proteome
Source: Cell Rep. Author manuscript; Available in PMC 2020 Jan 15. (PMC6961840; doi:10.1016/j.celrep.2019.11.026)
Supplement: 2 [file NIHMS1546469-supplement-2.zip › DF1/PXD006675/PulmonaryArtery/PulmonaryArtery_14_PALLD_EDLLNNGQPIYWFK.pdf]

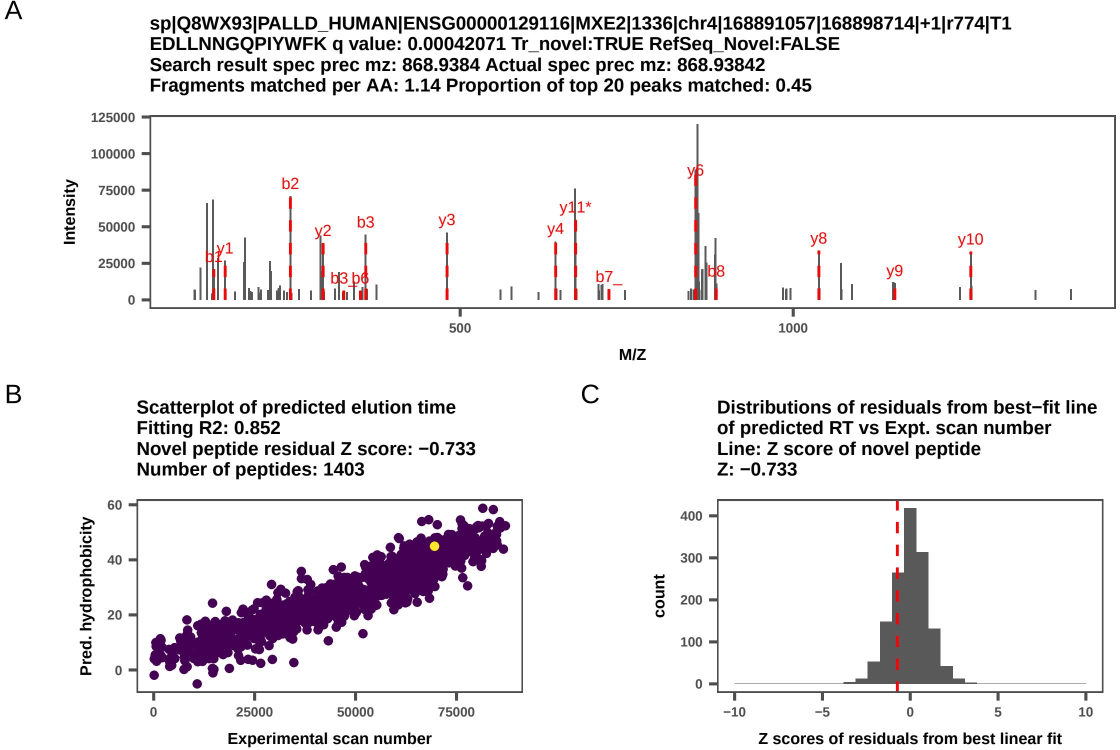
A
sp|Q8WX93|PALLD_HUMAN|ENSG00000129116|MXE2|1336|chr4|168891057|168898714|+1|r774|T1
EDLLNNGQPIYWFK q value: 0.00042071 Tr_novel:TRUE RefSeq_Novel:FALSE
Search result spec prec mz: 868.9384 Actual spec prec mz: 868.93842
Fragments matched per AA: 1.14 Proportion of top 20 peaks matched: 0.45
Intensity
125000
100000
75000
50000
25000
0
b1
y1
b2
y2
b3
b3_b6
y3
y4
y11*
b7_
y6
b8
y8
y9
y10
500
1000
M/Z
B
Scatterplot of predicted elution time
Fitting R2: 0.852
Novel peptide residual Z score: −0.733
Number of peptides: 1403
Pred. hydrophobicity
60
40
20
0
25000
50000
75000
Experimental scan number
C
Distributions of residuals from best−fit line
of predicted RT vs Expt. scan number
Line: Z score of novel peptide
Z: −0.733
count
400
300
200
100
0
−10
−5
0
5
10
Z scores of residuals from best linear fit
